# RNA multi-omics in single cells reveal rhythmical RNA reshaping during human and mouse oocyte maturation

**DOI:** 10.1186/s12915-025-02250-7

**Published:** 2025-05-28

**Authors:** Huan Yao, Danru Zhang, Haixia Jin, Yanjie Guo, Yan Liu, Shengnan Wang, Tong Li, Shenli Yuan, Gang Lu, Yingpu Sun

**Affiliations:** 1 Center for Reproductive Medicine, Henan Key Laboratory of Reproduction and Genetics, The First Affiliated Hospital of Zhengzhou University, Zhengzhou, China; 2 CUHK-SDU Joint Laboratory On Reproductive Genetics, School of Biomedical Sciences, The Chinese University of Hong Kong, Ma Liu Shui, Hong Kong; 3 CAS Key Laboratory of Genomic and Precision Medicine, Beijing Institute of Genomics, Chinese Academy of Sciences and China National Center for Bioinformation, Beijing, China; 4 GMU-GIBH Joint School of Life Sciences, The Guangdong-Hong Kong-Macao Joint Laboratory for Cell Fate Regulation and Diseases, Guangzhou Medical University, Guangzhou, China

**Keywords:** RNA terminal structure detection, RNA modifications, RNA multi-omics in single cells, Oocyte maturation, Maternal RNA decay

## Abstract

**Background:**

Omics technologies are widely applied in assisted reproductive technology (ART), such as embryo selection, investigation of infertility causes, and mechanisms underlying reproductive cell development. While RNAomics has shown great potential in investigating the physiology and pathology in female reproductive system, its applications are still not fully developed. More studies on epitranscriptomic regulation mechanisms and novel sequencing methods are needed to advance the field.

**Results:**

Here, we developed a method named Cap to Tail sequencing application (C2T-APP) and simultaneously characterized the m^7^G cap, poly(A) tail structure, and gene expression level for the intact RNA molecules in single cells. C2T-APP distinguished the N6, 2′-O-dimethyladenosine modification (m^6^A_m_) from *N*^6^-methyladenosine (m^6^A) modification with our published single-cell m^6^A sequencing (scm^6^A-seq) data. During oocyte maturation, we found a positive correlation of m^7^G and m^6^A_m_ with translation efficiency and finely dissected the step-wised maternal RNA de-capping and de-tailing of different types of genes. Strikingly, we uncovered a subtle structural mechanism regulating poly(A) tails in oocytes: maternal RNA translation is temporarily suppressed by removing the poly(A) tails without complete degradation, while the poly(A)-tail regulators themselves depend strictly on translation initiated after meiotic resumption. Furthermore, we profiled single-cell RNA-multi-omic features of human oocytes with different qualities during in vitro culture maturation (IVM). Defects of epi-transcriptome features, including m^6^A, m^6^A_m_, m^7^G, and poly(A) structure of maternal RNA in the oocytes with poor quality, were detected.

**Conclusions:**

Our results provided a valuable tool for RNAomics research and data resources provided novel insights into human oocyte maturation, which is helpful for IVM and oocyte selection for ART.

**Supplementary Information:**

The online version contains supplementary material available at 10.1186/s12915-025-02250-7.

## Background

Oocyte quality is one of the most crucial factors that influence the clinical result of assisted reproductive technologies (ART) [1]. Maternal RNA metabolism during oocyte maturation includes a series of processes accompanied by the changing of translation efficiency [2–5], location [6–8], and clearance [9] of maternal RNAs. Recent studies also highlighted the importance of maternal RNA metabolism fate regulation by RNA modifications [10–13] and poly(A) structure [14] for oocyte quality. Maternal RNA metabolism plays a critical role in early embryonic development, as it provides the necessary instructions for the first few cell divisions before the embryo switches to genomic transcription. Understanding maternal RNA metabolism during oocyte maturation is essential for elucidating the mechanisms of reproductive biology, embryonic development, and ART.

RNA metabolism is quite a complex process, and there are many different methods to profile the details of RNA metabolism during its life cycle, including transcription, splicing, translation, de-caping, and deadenylation [15]. The m^7^G cap structure on the RNA molecular infers the transcription initiation, while the 3′ poly(A) tail infers the transcription termination for most mRNAs in eukaryotes. Since the development of cDNA cloning, many distinctive RNA-seq methods have been developed to detect the 5′ termini and 3′ termini structure of RNAs [16, 17], such as CAGE-seq [18] and Tail-seq [19]. However, these methods usually require a large amount of RNA, which is imitated when using them on precious and rare samples such as mammal gametes and early embryos. For this purpose, some specialized RNA-seq methods have been developed to count the gene expression using 5′ or 3′ of the RNA at single-cell resolution [20, 21]. Both the cap and tail sequence can be captured in a single cell [22]. There has also been some impressive progress in profiling the poly(A) structure at the single-cell level [14, 23]. But to our knowledge, there is no short-read-based method to detect RNA molecules with intact information, including cap, tail structure, and gene body, in a single RNA-seq experiment.

During oocyte maturation, when the gene transcription is silenced, the metabolism of maternal RNA is completely controlled by the post-transcriptional elements on the RNAs. The most significant change in the maternal RNA is the largely decreased total and poly(A) RNA amount during oocyte maturation [24]. The RNA clearance process has been well studied using poly(A)-based single-cell RNA-sequencing methods [9, 23]. Many poly(A) tail regulation factors have been identified. Until now, most of these factors control maternal RNA clearance through the carbon catabolite repression 4-negative on TATA-less (Ccr4-Not) complex mediated deadenylating pathway [25, 26]. However, other RNA decay pathways such as RNA de-capping were largely unknown.

Recently, the m^6^A landscape has been studied [11, 12, 27] and the complexity of m^6^A modification in regulating maternal RNA decay has triggered new interest in how m^6^A works for maternal RNA metabolism during oocyte-to-embryo transition (OET). It is now known that N6, 2′-O-dimethyladenosine (m^6^A_m_), and m^6^A can play different roles in regulating RNA metabolism, and they are catalyzed by different enzymes [28]. The m^6^A_m_ is reported to regulate translation and stress response [29, 30], while the m^6^A plays many different roles through different binding proteins [31]. However, m^6^A_m_-seq requires a large amount (100 µg) of RNA sample [32] and current pipeline cannot distinguish m^6^A_m_ from m^6^A from m^6^A-seq data without m^7^G cap read identification, so the m^6^A_m_ is unknown in oocytes and early embryos. What is more, there is no data of the m^6^A atlas during human oocyte maturation due to technical challenges.

Due to its intrinsic terminal transferase activity, the Moloney murine leukemia virus (MMLV) reverse transcriptase adds a few cytosine (C) residues to the 5′ end of the newly synthesized first-strand cDNA during reverse transcription. This feature allows the enzyme to switch templates from the original RNA to a specially designed template switching oligo (TSO). This theory has been widely used for low-input mRNA library preparation protocols [33]. More interestingly, studies showed that the template switching works both at the m^7^G-cap 5′ termini RNA and non-cap 5′ termini RNAs, but the efficiency is influenced by the 5′ sequence and cap structure [34, 35]. Template-switching reaction was used to sequence the 5′ m^7^G cap of intact mRNA molecular [16], but it is unknown if the m^7^G cap can be identified from RNA-seq data starting from pre-fragmented RNA samples.

Beyond gene mutation profiling using whole exome sequencing (WES), cutting-edge epi-genomics [35] and proteomics [36] have been reported to be an important tool to study the failure of ART. The clinical application of RNA multi-omics is still in its early stages and requires more theoretical and methodological research. Previously, we reported scm^6^A-seq [12], which enables the profile of the m^6^A methylome and transcriptome at a single-cell level. Here, we utilize scm^6^A-seq to reveal the m^6^A landscape during human oocyte maturation. More interestingly, we performed a novel framework to understand the scm^6^A-seq data after careful and in-depth research. We named this improved analysis pipeline for RNA-seq as Cap to Tail sequencing application (C2T-APP). C2T-APP enables the performing of a very comprehensive analysis of the RNA molecular, including 5′ termini structure (m^7^G, m^6^A_m_) and 3′ termini structure (poly(A) length, uridylation), as well as gene expression level from scm^6^A-seq data. Using C2T-APP, we systematically analyzed both the total RNAome and the poly(A)ome in the same oocytes. What is more interestingly, we studied multi epi-transcriptome-omics of the maternal RNA from scm^6^A-seq data, including m^7^G, m^6^Am, m^6^A, and poly(A) length during oocyte maturation, including both mice and humans. These findings offer valuable RNAomics resources and into human oocyte maturation, which may provide novel insights in diagnosis and alternative treatments for oocyte quality in reproductive disorders.

## Results

### Development of C2T-seq

**Fig. 1:**
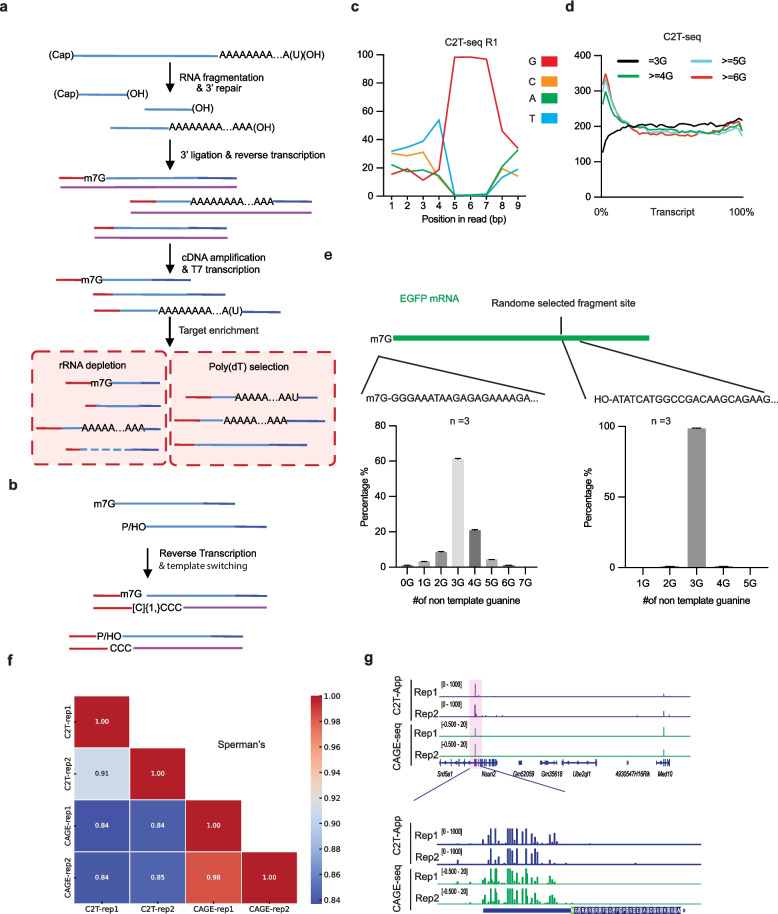
m^7^G-cap identification from RNA-seq data. **a** Schematic diagram showing the extra information in the RNA-seq libraries constructed using RNA-ligation-based methods such as scm^6^A-seq. We named this method C2T-seq. **b** Schematic diagram showing the computational model of different cDNA terminal structures due to the RNA 5′ modifications. **c** The first nine bases of the R1 data of C2T-seq libraries. **d** Meta-gene plot showing the coverage of different classes of reads, the reads are classified using the R1 reads of C2T-seq libraries. **e** A commercial EGFP mRNA with m7G cap and 100–150 bp poly(A) tail was used to confirm the different cDNA end of 5′ m7G RNA and OH/Phos 5′ structure RNA (upper). A random internal site was selected for further comparison (*n* = 3). Bar plot showing the number of G in the cDNA end of 5′ m7G RNA and OH/Phos 5′ structure RNA (lower). **f** Heat map showing the Spearman’s correlation value between the m^7^G sites level identified using CAGE-seq or C2T-seq App. **g** Integrated Genomics Viewer (IGV) diagram showing representative m^7^G sites identified by C2T-APP or CAGE-seq. The zoomed-in view below highlights that the m.^7^G sites detected by both methods exhibit very close alignment at single-base resolution (below)

RNA sequencing (RNA-seq) is one of the most important technologies in genomics. Mature mRNAs typically contain a 5′ cap and a 3′ poly(A) tail; however, existing RNA-seq methods often fail to fully capture these features, resulting in incomplete representation of the mRNA molecules. To address this issue and develop a method capable of capturing the complete information of mRNA, we summarized several commonly used single-cell RNA-seq protocols and identified coverage biases introduced during first-strand and second-strand synthesis steps: in a standard RNA-seq protocol, the RNA was reverse transcripted into cDNA, before the second-strand cDNA was synthesized by RNase H and DNA polymerase. However, this protocol causes the loss of RNA information near the 3′ termini during cDNA synthesis and the 5′ termini during second-strand synthesis (Additional file 1: Fig. S1a, d). For the SMARTer Stranded Total RNA-Seq Kit and SMARTer smRNA-Seq, the random priming step leaves a sequencing margin near the 3′ termini of RNA, the polyadenylation step for cDNA synthesis causes a loss of the poly(A) tail length information, and the reverse transcription termination before 5′ cap causes partly loss of m^7^G signal (Additional file 1: Fig. S1b). For smart-seq, the reverse transcription using oligo(dT) causes the loss of poly(A) length information and the library preparation step causes a loss of both sequences near the 5′ and 3′ termini for smart-seq2 (Additional file 1: Fig. S1c). Further analysis showed a common defect of capturing the RNA information near the cap and tail structure (Additional file 1: Fig. S1 d). For scm^6^A-seq, the RNA is randomly fragmented before the 3′ termini was ligated with adaptors. The 5′ termini are reverse transcripted into cDNA during the template-switching reaction. As a result, scm^6^A-seq can capture both the 3′ termini and 5′ termini information of the RNA molecular. To enrich the poly(A) information, we added a poly(A) selection step after the reverse transcription of the library using oligo(dT) beads (Fig. 1a). We named this workflow to prepare RNA-seq libraries as Cap to Tail sequencing (C2T-seq).

### *m*^*7*^*G cap identification from C2T-seq*

As m^7^G increases the efficiency of template-switching reaction [34, 35], we proposed that the m^7^G can be reverse transcripted into cDNA (Fig. 1b). And we observed a bias of base distribution at the first site of cDNA (Fig. 1c), then we separate the reads into different classes according to the sequences near the TSO. Strikingly, we found the coverage bias increases near the transcription start site (TSS) in the subset sequencing data starting with several G bases following the TSO sequence (Fig. 1d). Interestingly, we also observed an enrichment of TSS in the ≥ 4G subset sequencing data from SMARTer Stranded Total RNA-Seq Kit (Additional file 1: Fig. S1e). This result suggests that the m^7^G modification promotes template switching by inducing more G base at the 3′ end of cDNA, which helps the TSO priming during reverse transcription reaction through C-G pairing. Then, we sequenced the commercial EGFP mRNA with C2T-seq. As expected, we found a significant difference in the distribution of non-template G numbers at the m^7^G cap and internal random selected site (Fig. 1e upper). The m^7^G structure causes a flexible template-switching result. More than 20% of RNA molecular with a m^7^G cap were reverse transcripted into cDNA with more than three G at the end of cDNA, while almost all cDNA from RNA molecular with hydroxyl (OH) have strictly three G at the 5′ termini (Additional file 1: Fig. S1e lower).

To further validate our finding, we compared C2T-seq with CAGE-seq data and inter sample comparation is also performed. When we compared the m^7^G site at single base resolution, we found 51.5% m^7^G sites of CAGE-seq is repeatable in both repeats, and that value is 34.7% for C2T-APP (Additional file 1: Fig. S2a upper), C2T-APP identified 18,947 m^7^G sites from RNA-seq data, while CAGE-seq identified 97,447 m^7^G sites. More than half m^7^G sites is in the CAGE-seq data (Additional file 1: Fig. S2a bottom left), and no random sites in the CAGE-seq data identified m^7^G sites (Additional file 1: Fig. S2a bottom right). For reproducibility analysis, the correlation value between two repeats of C2T-seq is 0.91 (Additional file 1: Fig. S2b). We also find a strong correlation between CAGE-seq and C2T-APP (Fig. 1f), And the global correlation value is between 0.84 and 0.85. All these results strongly suggest that C2T-APP identified the m^7^G-cap using RNA-seq data with a high accuracy.

### C2T-APP counts RNA molecular from cap to tail

**Fig. 2:**
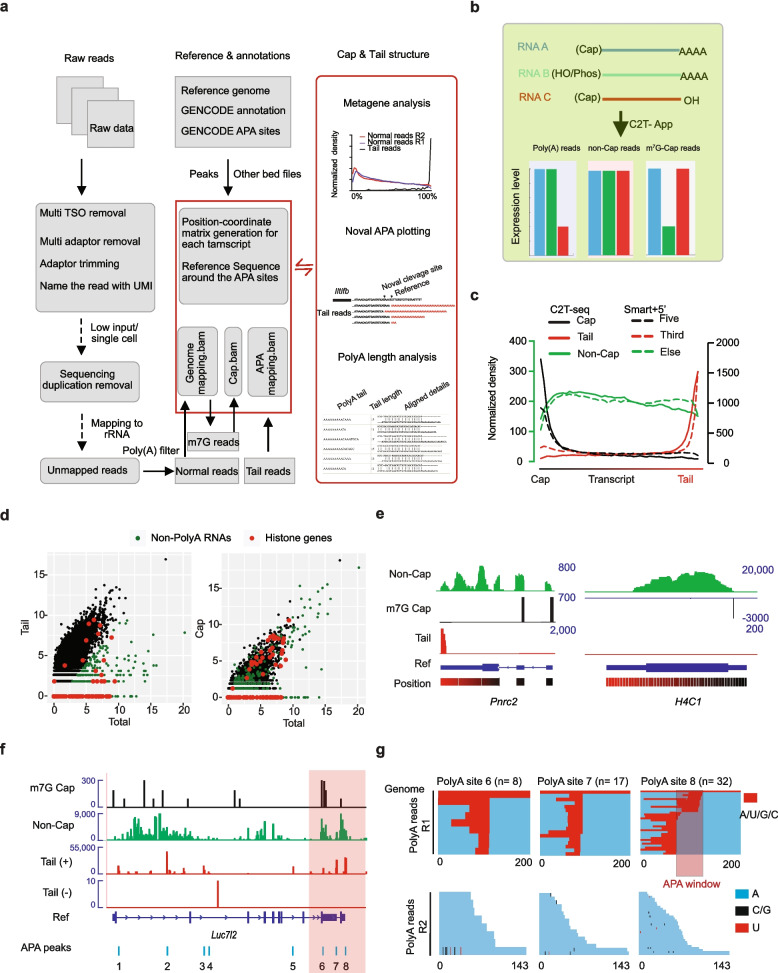
RNA multi-omics analysis using C2T-APP. **a** Bioinformatic analysis flowchart of C2T-seq data, we named this pipeline as C2T-APP. **b** Schematic diagram illustrating the C2T-APP method for simultaneous multi-dimensional quantification of RNA expression levels based on cap structures, poly(A) tails, and gene bodies. **c** Meta-gene plot of the cap reads, tail reads, and non-cap reads separated from a single RNA-seq data using C2T-APP or Smart-seq + 5′. **d** C2T-APP identified the poly(A)-minus and m7G-cap minus genes. Dot plot showing the different gene expression levels using total reads and tail reads (left), total reads and cap reads (right). **e** Integrated Genomics Viewer (IGV) diagram showing the distribution of the cap reads, tail reads, and non-cap reads of presentative poly(A)-plus mRNA *Pnrc2* (left) and poly(A)-minus histone RNA *H4c1* (right). The relative position reference of the is labeled by a color bar (black to red). **f**–**g** Integrated Genomics Viewer (IGV) diagram showing the distribution of the cap reads, tail reads, and non-cap reads of a presentative gene Luc712. The identified APA sites are shown below the gene reference (**f**). Three presentative tail reads are shown by aligned to the genome for R1, and the poly(A) length is shown by R2. The largest distance among the observed A-tailing sites within the same APA site is heightened (**g**), and we named this the APA window

Then, we established a novel pipeline to analyze the C2T-seq data (Fig. 2a). After standard read processing according to scm^6^A-seq [12], we extracted the poly(A) read from the first sequences in R2 read. Then, the other reads are mapped to the genome before the m^7^G reads are extracted according to the number of non-templated G. Then, the data were individually analyzed to quantify gene expression derived from m^7^G-capped reads, gene body reads, and 3′ tail reads (Fig. 2b). This enables us to calculate gene expression from 5′ m^7^G or 3′ poly(A) terminal using one RNA-seq data. Then, we applied C2T-APP to analyze bulk RNA-seq data of the mESC cell line. Our data shows that C2T-APP extracts both the cap reads, tail reads, or geneBody reads accurately (Fig. 2c, solid lines). Furthermore, we found C2T-APP achieved similar results as the latest Smart-seq + 5′ without modifying the process of RNA library construction steps [37] (Fig. 2c, Additional file 1: Fig. S1f). As expected, C2T-APP identified the non-poly(A) transcripts (Fig. 2d left) and found many genes without poly(A) structure (Fig. 2e), such as histone genes [38]. What is more, we also found there are many histone genes without m^7^G cap structure (Fig. 1d right). These results proves that C2T-seq provides a better understanding of the total transcriptome.

### Alternative polyadenylation sites (APA), poly(A) length, and modifications in the 3′ UTR profiling with C2T-seq

Then, we further performed APA sites analysis using the poly(A) signal reads (Additional file 1: Fig. S2c). Firstly, we trimmed the continuous “A” base from R1 data and discarded the reads shorter than 18 bp. While most methods for poly(A) length analysis fragment the RNA using RNase T1, C2T-seq performs RNA fragmentation by heating the RNA with the appearance of Mg2 +. To reduce the impact of library size [19] and potential RNA fragmentation inside the poly(A) tail for poly(A) length calculation, the perfectly merged reads (less than 300 bp) were discarded [39], and only the reads longer than 300 bp were left for further analysis. Then, the R1 reads were mapped to the genome, and the APA peaks were called using macs2. Then, we compared our APA peaks with the published APA sites database and identified 4621 highly confident APA sites. Our data showed significant enrichment of poly(A) reads around the − 100 to + 100 bp regions of reference APA sites compared to randomly selected regions (Additional file 1: Fig. S2f). More interestingly, C2T-seq detected many novel APA sites not presented in the published database, and we identified many APA sites in the coding sequences (CDS) (Fig. 2d). For mESC transcriptome, while most genes had a single APA site, the most comprehensive gene codes many isoforms with 8 APA sites (Additional file 1: Fig. S2e), such as Luc7 l2 (Fig. 2f). What is more interesting is that by aligning the poly(A) reads with the genome sequences within the APA peaks, we found several polyadenylation sites in the same APA peak region. Then, we defined the largest distance between the APAs inside an APA peak as an APA window (Fig. 2g). Further analysis showed the APA windows distance distributed in 1–40 bp, and most APA windows are less than 10 bp (Additional file 1: Fig. S2 g). For poly(A) length analysis, the C2T-APP trimmed the R2 data by sequencing quality and base content. Our result showed the average poly(A) length of mESC transcriptome as 55–75 bp for most transcripts (Additional file 1: Fig. S2 h). This result is consistent with previously reported mammalian poly(A) length [19]. What is more impressively, we separated the u-tail poly(A) reads from other poly(A) reads and found a significantly shorter poly(A) tail in the U-tail modified transcripts (Additional file 1: Fig. S2i). These findings demonstrate that the information of poly(A) length is preserved in libraries constructed using the C2T-seq protocol, and C2T-APP profiles the APA sites at single base resolution.

### *C2T-APP identifies m*^*6*^*A*_*m*_* from scm*^*6*^*A-seq data*

**Fig. 3:**
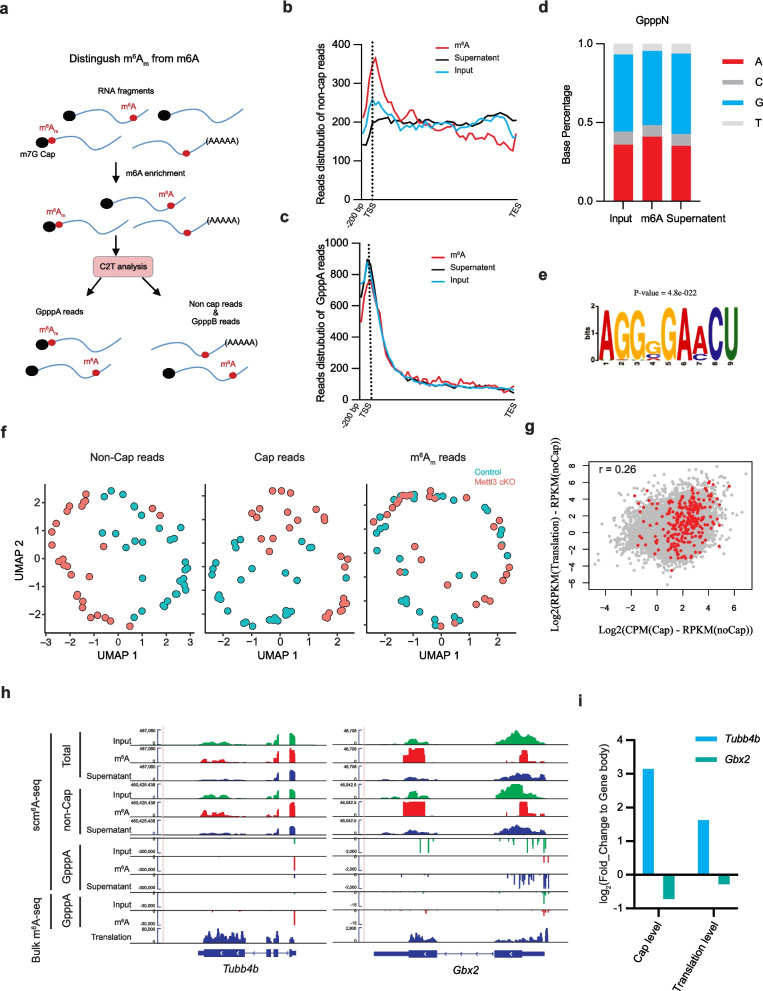
m^6^A_m_ and m^7^G cap improves maternal RNA translation in the oocytes. **a** Schematic diagram showing the principle of C2T-APP distinguishes m^6^A_m_ from m^6^A from scm^6^A-seq data. Meta-gene plot of m^6^A-IP, input, and supernatant reads of non-cap reads (**b**) and m^7^G-cap reads (**c**). **d** Bar plot showing the base content distribution next to the identified m^7^G site by m^6^A-IP, input, or supernatant data set. **e** The enriched motif of the RNA base content down steam of the identified m^6^A_m_ sites.** f** UMAP plot of the WT (*n* = 31) and cKO (*n* = 31) oocytes by either non-cap reads (left), m^7^G-cap reads (middle), or m^6^A_m_ reads (right). **g** Dot plot showing the positive correlation (*r* = 0.26) of m^7^G-cap/m^6^A_m_ level and translation efficiency in GV oocytes. The m^6^A_m_ modified genes are in red. **h** Integrated Genomics Viewer (IGV) diagram showing the multi-omics result of *Tubb4b* (with m^6^A_m_) and *Gbx2* (without m^6^A_m_) gene expression in the oocytes by scm^6^A-seq data. Bulk m^6^A-seq data was also shown below. **i** Bar plot showing the relative m.^7^G-cap level and translation level of *Tubb4b* and* Gbx2*

As C2T-APP recognizes the m^7^G reads, we wonder if C2T-APP can be used to profile the m^6^A_m_ from scm^6^A-seq data. Because the m^6^A_m_ appears with an m^7^G cap in the RNA [40], and the anti-m^6^A antibody can bind both the m^6^A_m_ and m^6^A [41]. So, we separated the scm^6^A-seq data into different parts and called the m^6^A_m_ from m^7^GppA reads (Fig. 3a). Then, we applied this pipeline to the data set of our previously reported scm^6^A-seq data of mouse oocytes [12]. Firstly, we separated the m^7^GpppA reads and found a significant enrichment of m^7^GpppA reads at the TSS sites compared to non-cap reads in scm^6^A-seq data and bulk m^6^A-seq data (Fig. 3b–c, Additional file 1: Fig. S3a–b). There was an increase of m^7^GpppA reads in the m^6^A immunoprecipitation (m^6^A) dataset compared to input and supernatant data (Fig. 3d, Additional file 1: Fig. S3c). Then, we used the 20 bases after m^7^G to find the m^6^A_m_ motif. We found the most significant motif is “AGGVGGRACU,” which starts from A flowed by several G. This result suggests that the G in the TSS may contribute to the catalyzing of m^6^A_m_, and the GRACH in the motif is consistent with the previous report [41]. Then, we analyzed the m^6^A_m_ at a single-cell level. As expected, the single-cell expression matrix calculated using non-cap reads or cap reads can distinguish the WT and *Mettl3* cKO oocytes (Fig. 3f left and middle). However, we did not find a significant difference in m^6^A_m_ (Fig. 3f left and middle). This result suggested that the m^6^A_m_ is less affected by the gene expression level. What is more, this result showed that the m^6^A_m_ is not affected by the knocking out of *Mettl3* in the oocytes because it is catalyzed by *Pcif1* but not *Mettl3* [32, 42]. Then, we aggregated the WT and *Mettl3* cKO data for further analysis. For m^6^A_m_ calling, we identified 587 m^6^A_m_ genes in scm^6^A-seq data and 1613 m^6^A_m_ genes using bulk m^6^A-seq data (cutoff, m^7^GpppA (IP) − m^7^GpppA (input) > 0.5). Then, we defined the m^6^A_m_ genes that existed in both scm^6^A-seq and bulk m^6^A-seq as highly confident m^6^A_m_ genes (Additional file 1: Fig. S3 d). GO analysis showed that the most significant enriched functions of m^6^A_m_ genes in the oocytes are related to stress response, which is consistent with previous work [43] (Additional file 1: Fig. S3e). Maternal RNA translation is the most important event in the oocytes, and the translation initiation is largely regulated by the m^7^G cap [44, 45]. Then, we defined the m^7^G cap level using log_2_ [CPM (Cap)/CPM (nonCap)] and the translation efficiency using published translatome data [3] as log_2_ [CPM (Translation)/CPM (nonCap)]. As expected, our data suggests a moderate but overall positive correlation (*r* = 0.26) between m^7^G cap level and translation efficiency (Fig. 3g), and this result suggests the role of m^7^G-dependent translation control in the oocytes. What is more interestingly, we also found that the m^6^A_m_ modified transcripts had a higher correlation coefficient with the translation efficiency (Additional file 1: Fig. S3f). Then, we further confirmed the role of m^6^A_m_ in promoting RNA translation by separating the genes into different classes according to their m^7^G level. The result showed that most m^6^A_m_ genes (2 < m^7^G ≤ 4) translated significantly higher than other genes (Fig. 3h–i, Additional file 1: Fig. S3 g). This result is consistent with the previously reported result that “m^7^GpppA_m_pG” RNA was the fastest translated [46]. These findings demonstrate that C2T-APP leads to a much deeper understanding of scm^6^A-seq data.

### Maternal RNA clearance is translation-dependent

**Fig. 4:**
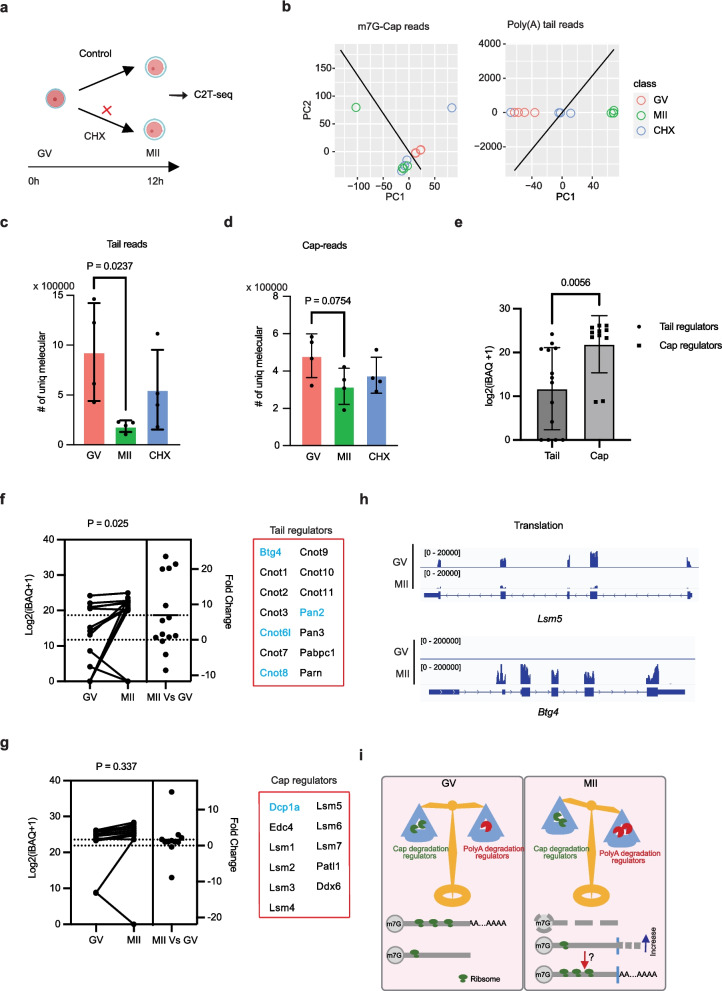
Maternal RNA poly(A)-tail structure regulates translation efficiency reshaping during oocyte maturation. **a** Schematic diagram showing the experimental design of translation inhibition during oocyte maturation. **b** Dot plot showing principal component analysis (PCA) result of C2T-seq result of individual oocytes by m^7^G-cap reads and poly(A) reads. The line shows the best support vector machine (SVM) classification result of GV oocytes and MII oocytes. Bar plot showing the poly(A)-reads (**c**) and cap reads (**d**) detected in the GV oocytes (*n* = 4), MII oocytes (*n* = 4), and CHX-treated MII oocytes (*n* = 4). *T*-test was used to detect the different RNA content in GV oocytes and MII oocytes. **e** Bar plot showing the expression levels (log2(iBAQ + 1)) of poly(A) tail regulators and m^7^G-cap regulators in oocytes, as determined by mass spectrometry analysis of public data [2]. Statistical significance (*p* value = 0.0056) was calculated using a two-tailed *t*-test. Estimation plots illustrating the expression levels (log2(iBAQ + 1)) and corresponding fold changes of poly(A) tail regulators (**f**) and m^7^G-cap regulators (**g**) between GV and MII oocytes. Genes exhibiting the most significant changes are highlighted in blue. Statistical significance was assessed using a paired two-tailed *t*-test. **h** Integrated Genomics Viewer (IGV) diagram showing the different translation level of cap regulator LSM5 and BTG4 in GV oocytes and MII oocytes. **i** Cartoon diagram illustrating how poly(A) and cap regulators control maternal RNA metabolism and translation in GV and MII oocytes. Following meiotic resumption (GV to MII transition), poly(A) degradation regulators are rapidly translated and accumulate in oocytes, leading to reduced translation of maternal RNAs through poly(A) tail removing, while the relative lack of m^7^G-cap degradation regulators preserve certain poly(A)-minus maternal RNAs. Since their gene bodies remain intact, these RNAs may be re-polyadenylated and retranslated during subsequent developmental stages

Maternal RNA clearance and translation are the most important issues during oocyte maturation, so we wonder if there is any connection between maternal translation and clearance. We cultured the GV oocytes with a translation inhibitor cycloheximide (CHX). As expected, the maturation ratio decreased (Fig. 4a, Additional file 1: Fig. S4a–b). What is more interesting is that we found the metabolism of the cap was less affected than that of poly(A). When calculating the gene expression matrix using m^7^G or gene body data, 75% of the MII oocytes cultured with CHX are classified into the same class as MII. But the 75% MII oocytes cultured with CHX are classified into the same class with GV when calculated using poly(A) or poly(A) length matrix (Fig. 4b, Additional file 1: Fig. S4c). Further analysis showed that the maternal RNA deadenylation was more affected in the CHX-treated MII oocytes than the m^7^G cap and gene body (Fig. 4c, d, Additional file 1: Fig. S4 d). To validate our findings, we analyzed the proteome data of GV oocytes [47]; strikingly we found a significant higher level of m^7^G regulators than poly(A) regulators in the GV oocytes (Fig. 4e). And the protein level increased significantly in the MII oocytes compared to GV oocytes (Fig. 4f). But most of the m^7^G regulators keep stable during oocytes maturation except Dcp1a (Fig. 4g). The translation data also suggests that most m^7^G regulators such as *Lsm5* decrease their translation (Fig. 4h upper) while poly(A) regulators accumulated rapidly by increased their translation during the process of oocyte maturation such as *Btg4* (Fig. 4h bottom). This result suggests that most maternal RNA lost poly(A) during oocyte maturation, but the RNA is not degraded completely, which supports that the maternal RNA re-polyadenylation later during embryo development [14].

### The translation efficiency is regulated by the 3′ poly(A) tail structure during oocyte maturation

The mechanism of maternal RNA metabolism fate has been one of the most important issues for maternal RNA metabolism during maternal RNA maturation. Previous research has revealed different speeds of RNA decay and translation [3, 9, 26]. To further classify the maternal RNA into different types, we combined the maternal RNA expression matrix calculated using cap reads, tail reads, and gene body reads of both GV and MII samples. Very interestingly, the maternal RNAs were separated into four classes (Additional file 1: Fig. S5a). Then, we identified class 1 as stable genes, class 2 as lost poly(A) genes, and class 3 and class 4 as non-poly (A) tail GV transcripts after further analysis: the class 4 and class 3 genes have lower expression level in poly(A) data than gene body data (Additional file 1: Fig. S4e). The class 2 genes are greatly decreased in poly(A) data (Additional file 1: Fig. S4f), and the poly(A) degradation is translation-dependent (Additional file 1: Fig. S4 g).

What is more strikingly, our data revealed a decreased translation efficiency for 3′ poly(A) minus transcripts in both GV and MII samples (Additional file 1: Fig. S5b, Additional file 1: Fig. S4 h). Then, we also summarized the relationship between translation efficiency changes during oocyte maturation using the translation efficiency changed genes (TE(MII) − TE(GV) > 2 or TE(MII) − TE(GV) <  − 3). Our results showed a positive correlation between poly(A) structure and cap structure level with translation efficiency during GV to MII transition (Additional file 1: Fig. S5c, e). We also did a GO analysis of the poly(A) controlled translation dynamic genes and found a significant enrichment of genes relative to cell division and chromatin remodeling for translation-increased genes, and ribonucleoprotein complex biogenesis and mitochondrial relative genes for translation decreased genes (Additional file 1: Fig. S5 d).

Next, we wonder if the poly(A) structure was related to RNA gene body degradation in the oocytes. Then, we identified the most dynamic RNAs using gene body data. Our data suggests there is no significant correlation between poly(A) structure clearance and gene body degradation. Poly(A) length analysis also showed no significant shortening of the poly(A) length for the unstable transcripts (Additional file 1: Fig. S4j–k). All these results suggests that maternal poly(A) regulators digest the poly(A) structure of maternal RNA to decrease the translation during oocyte maturation, due to the lack of m7G remove factors, the non-poly(A) transcripts can be stored for further usage for translation (Fig. 4i).

### Multi-RNAome profiling of human oocytes of different quality

**Fig. 5:**
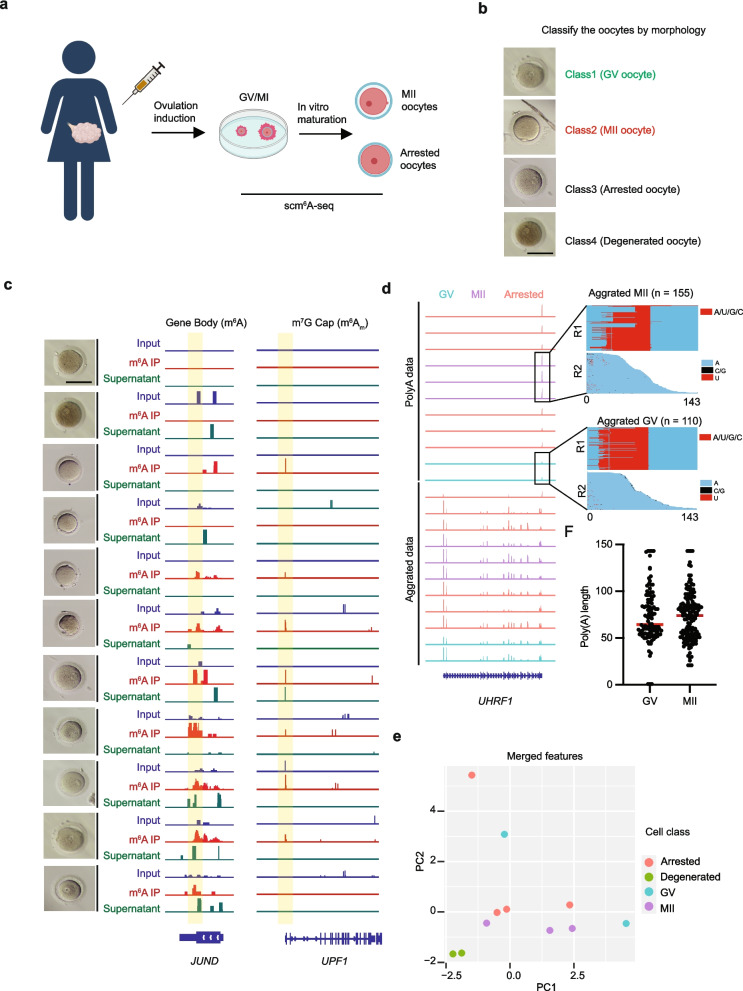
scm^6^A-seq reveals a single-cell multi-omics RNAome landscape of human oocytes of different qualities. **a** Schematic diagram showing the experimental design of human oocyte collection and culture for scm^6^A-seq. **b** Presentative morphological image showing the collected human oocytes of different qualities, the black scale bars represent 100 μm. **c**–**d** scm^6^A-seq simultaneously captured m^6^A, m^6^A_m_, gene expression level, m^7^G level, and poly(A) gene expression level of human oocytes at single-cell level. Integrated Genomics Viewer (IGV) diagram showing the mapping result of m^6^A, m^6^A_m_ of two presentative genes of human oocytes individually (**c**) and poly(A) gene expression level with poly(A) tail length of *UHRF1* gene were shown right beside (**d**). The GV oocytes, MII oocytes, and translation-inhibited MII oocytes were shown in different colors; each oocyte’s morphology is shown on the left (**c**). The black scale bars represent 100 μm. **e** Dot plot showing the poly(A) tail length of the *UHRF1* gene in human GV and MII oocytes. The red line shows the median value of the poly(A) tail length. **f** Principal component analysis (PCA) analysis of the multi-omics matrix of the RNAome data of each oocyte. The cells were classified by the morphological photos shown in **b** with the distance among each cell in the PCA distance matrix

To study the details of RNA metabolism during human oocyte maturation, we collected the unmatured human oocyte which was arrested at different stages (Fig. 5a). The oocyte morphology was recorded before cell lysis (Fig. 5b). Then, we sequenced thirteen oocytes using scm^6^A-seq. For data qualifying, we also sequenced two granule cell clusters (50–100 cells) at the same time (Additional file 1: Fig. S6a upper). Using C2T-APP, we profiled the m^7^G, m^6^A, m^6^A_m_ gene expression (Fig. 5c), poly(A)ome, and tail length (Fig. 5d) from the same oocyte. The data quality was further proved by the significantly distinguished oocytes and granule cell clusters by any part of the RNA features (Additional file 1: Fig. S6b–f). Poly(A) tail length calculation were detected using PE150 mode to exclude the potential loss of poly(A) tail more than 143 bp, which is the longest length. We sequenced the poly(A) enriched library using both PE150 and PE250 modes, and our results showed that most poly(A) tail is less than 143 bp (Additional file 1: Fig. S6 h). Our results showed similar results of poly(A) length between PE150 data and PE250 data of both aggerated data and single-cell data (Additional file 1: Fig. 6 h–i). So, we used only the poly(A) data sequenced under PE150 mode for further analysis. What is more, the correlation of poly(A) data of the same oocyte (PE150 vs PE250, *r* = 0.38) is higher than that of oocytes in different stages (GV vs MII, *r* = 0.28) (Additional file 1: Fig. 6j). These results showed that C2T-APP could capture multi-omics features of the RNA from scm^6^A-seq data at the single-cell level. Then, we combined the top 2 levels of PCA matrixes of each RNA feature and performed a hierarchical clustering using the combined matrix (Additional file 1: Fig. S6 g). A further PCA was performed on the combined PCA matrix and the combined RNA molecular features distinguish the oocytes in different morphology (Fig. 5e). But scm^6^A-seq also reveals many differences between oocytes with similar morphology features. For the class oocytes with germinal vesicles, the RNA features are very different from other GV oocytes. By a further summary of these two cells, we found a phenotype of dark-yellow cytoplasm of class 4 cells, which conforms to the characteristics of degenerated oocytes according to our knowledge. All these results showed a high heterogeneity of RNA metabolism during oocyte maturation, and our data provided a new perspective for studying the nature of oocyte maturation arrest.

## Discussion

The difference among transcriptome, translatome, and proteome induced much interest in researching the translation regulation pattern in the oocytes [2, 14, 47, 48]. It is widely believed that the translation is regulated by the poly(A) polymerase, which elongates the poly(A) length of specific transcripts [42]. At the same time, RNA degradation is regulated by mRNA decay factors such as Btg4, which are translated after meiosis resumption [9, 26]. Recent studies also provided the importance of poly(A) (poly(A) length and APA sites) regulation during oocyte maturation and translation control [23]. What is more, the role of RNA methylation also emerged as a novel factor in maternal RNA regulation [11–13, 27, 49]. All these important advances have benefited from the development of technology and the integrated analysis of multi-omics data. However, most studies focused on the poly(A) plus subset transcriptome or part of the RNA molecular due to the limitation of sequencing methods. C2T-APP is another important tool for researching the process of RNA metabolism during oocyte maturation and early embryo development, and we successfully described a multi-omics RNA landscape including m^6^A, m^6^A_m_, cap and tail structure, poly(A) length, and gene expression for each gene of both mouse and human. Our data will be valuable for this field and help to explore new mechanisms of RNA metabolism during oocyte maturation and cell fate differentiation.

Here, we observed the process of RNA degradation and translation during oocyte maturation are coupled and mutually influencing, but the details and mechanism are still largely unknown. There are two hypnoses to explain this phenomenon. First, the RNA degradation factors such as Btg4 are at a low level until the translation firing after miosis resumption; this makes the maternal RNA hyper-stable in the translation-blocked oocytes, but this cannot explain the heterogeneous degradation patterns among different gene sets. Secondly, we hypothesized that the translation process may influence RNA degradation by reshaping the conformation of the mRNA molecular, such as RNA-RNA interaction [50]. Translation-dependent RNA decay by reducing the stability of mRNA was recently studied in cell lines [51], but the functions of this process during oocyte maturation are still unknown. We believe this is very interesting, but experiments are needed to clarify this hypnosis.

Although there are ATAC-seq data and Chip-seq data of multiple histone modifications and translation factors, it is necessary to confirm the activity of promoters and enhancers using CAGE-seq data. Recently, there were works to capture the enhancer activity during oocyte maturation and early embryo development using low-input CAGE-seq [52–54]. C2T-seq can identify the active cis-elements and can be used to identify the m^7^G level of a specific gene at a single-cell level. So, it will be interesting to explore the activity of promoters and enhancers during embryo development at single-cell level using C2T-APP.

Technically, compared to other 5′ termini RNA-seq data, which can detect the end of RNA transcripts, it cannot identify the activation of cis-elements as CAGE-seq dose because 5′ single-cell RNA-seq can only capture the poly(A)-tail transcripts, and there is a lack of methods to further analysis the m^7^G structure from 5′ single-cell RNA-seq data.

There are many efforts to detect m^6^A_m_ from m^6^A-seq data; some methods use CAGE-seq reference to annotate the m^6^A_m_ according to the distance of m^6^A peaks to the TSS site [55, 56]. However, these methods cannot identify the m^6^Am site precisely at single-cell base resolution because the TSS sites often appear in a region but not a single base. The specific TSS sites can be variable in different cell types or tissues. Here, the C2T-seq can identify the m^7^G-cap of the transcripts, which has largely resolved this difficult point.

Compared to the 3′ single-cell RNA-seq data, C2T-seq can detect the APA sites and the poly(A) length at the same time, and these features make a broad usage in the future. In C2T-seq, the tail reads are further enriched by poly(dT) oligos. Further work should be focused on the enrichment of cap reads. What is more, C2T-APP can also work on the libraries constructed using template-switching reactions. This highlights the importance and promotional value of our research method.

Limitation of this study: (1) the acquisition of oocytes is often limited by donor availability, which may affect the representativeness of the study. Additionally, human oocytes exhibit significant individual variability, including differences in genetic background, age, and physiological conditions, which may introduce experimental bias. Such variability could impact the reproducibility and generalizability of the results. Therefore, future studies should consider using larger sample sizes and integrating multi-omics approaches to better analyze the effects of individual differences. (2) This study provides an in-depth investigation of the multi-omics characteristics during oocyte maturation. However, the mechanistic insights remain relatively limited, as our analysis primarily focuses on omics-based associations. Future research will take a step further by focusing on key molecules involved in RNA metabolism and leveraging multi-omics approaches to elucidate both the observed RNAomic changes and their underlying molecular mechanisms. Specifically, studying RNA de-capping factors such as Dcp1a, the m^6^A_m_ methyltransferase Pcif1, and other RNA degradation regulators will significantly enhance our understanding of RNA metabolic regulation during oocyte maturation.

## Conclusions

In summary, our study developed a single-cell multi-omics sequencing method (C2T-seq) and an analysis framework (C2T-APP), which enable a more comprehensive analysis of RNA-omics data. Notably, the ability to directly identify m^7^G sites from transcriptomic data provides an important tool for epigenetic research. Additionally, this study mapped the multi-omics changes in RNA during the maturation of human and mouse oocytes through integrative RNA multi-dimensional analysis, offering a valuable resource for research on in vitro oocyte maturation.

## Methods

### Human oocyte collection

We collected oocytes from ICSI-treated patients who met the following criteria: age ≤ 35 years, neither spouse has any known genetic diseases. The ovarian stimulation was performed using the standard long protocol, and oocytes were retrieved 37 h after triggering. Only GV oocytes were collected when they were not matured to MII oocytes after 12 h of culture of the granulosa cell oocyte complex (COC) after standard treatment.

In order to ensure the quality of control oocyte samples, we cultured immature oocytes in clinical specialized in vitro culture medium and selected high-quality oocytes with morphology consistent with conventional mature oocytes as the control group for sequencing. The samples were collected for three times; we finally collected 11 oocytes for scm^6^A-seq analysis including at least two morphologically intact GV and MII oocytes.

### Animal

All mice in this study were kept at C57BL/6 J genetic background and housed under specific pathogen-free (SPF) conditions with 12 light/12 dark cycles at 22 °C, 40% humidity. All animal experiments were approved by the Animal Care and Use Committee of China Agricultural University and the First Affiliated Hospital of Zhengzhou University.

### Cell culture

The mouse embryo stem cells (mESC) were cultured in a cell culture dish coated with 0.1% gelatin and filled with high glucose DMEM (Invitrogen, C11995500BT), 20% fetal calf (bovine) serum (Invitrogen, 10099141 C), 2 mM L-glutamine (Gibco, 25030081), 1 mM sodium pyruvate (Gibco, 25030081), 100 μM non-essential amino acids (Gibco, 11140050), 0.1 mM 2-mercaptoethanol (Gibco, 21985), 50 U/ml penicillin–streptomycin (Gibco, 15070063), 3 μM CHIR99021, 1 μM PD0325901, and 10,000 units/ml LIF.

On the other hand, the 293 T cell line was cultured in DMEM containing 10% FBS. Both mESC and 293 T cells were cultured at 37 °C with 5% CO_2_.

### Library construction

As shown in Fig. 1a, C2T-seq libraries were crafted using the scm^6^A-seq protocol [12] without m^6^A enrichment. Instead, we utilized additional poly(A) tail RNA enrichment, which significantly enhances the accuracy and sensitivity of the detection of poly(A) plus transcripts of scRNA-seq data. Single cells were piped into individual tubes with 5 µl lysis buffer (0.5 µl of 10 × lysis buffer (TaKaRa, 635013), 2.5 µl 2 × FPE buffer (Vazyme #N402), 0.5 µl of 40 U/ml RNase inhibitor, murine (NEB, M0314L), 0.5 µl of gDNA eraser (CWBIO, HiFiScript gDNA Removal cDNA Synthesis Kit, CW2582M), 1 µl of nuclease-free water). Keep the samples at room temperature for 3 min for cell lysis before gDNA was removed by gDNA eraser at 42 °C for 5 min, then RNA was fragmented to about 200 bp by heating at 94 °C for 8 min. Then, gDNA eraser was deactivated by heating at 75 °C. Then, add T4 PNK and PNK buffer (NEB, M0201L) to each reaction at 37 °C for 45 min to remove RNA 3′ phosphate group before RNA adapter ligation. For the ligation reaction, 2 µl T4 ligation buffer (NEB), 1 µl T4 RNA ligase 2, truncated K227Q (NEB, M0373L), 7 µl 50% PEG8000 (NEB), 1 µl pre-adenylated adaptor, and 1 µl RNase inhibitor (NEB, M0314L) were added to each tube. (Note: Record the adaptor ID and samples’ information for downstream data processing). RNA ligation was performed at 4 °C overnight with shaking at 350 RPM speed. Then, RNA from all tubes was pooled together and purified using 1.5 × RNA Clean XP beads (Beckman, A63987). After that, the T4 RNA ligase is inactive at 75 °C for 20 min to stop ligation. After eluting RNA in 40 µl nuclease-free water, the template-switching reaction is performed using picoRT oligo and 5′ blocked TSO before cDNA purification using 1 × DNA clean beads and eluted in 32 µl NF water. Then, 40 µl 2 × KAPA HiFi HotStart ReadyMix (KAPA, KK2602), 4 µl pico-RT primer, and 4 µl T7-pre-universal primer are added. The first PCR amplification was carried out using this program: 98 °C for 1 min, 72 °C for 1 min, 60 °C for 1 min, 72 °C for 2 min, 98 °C for 1 min, 14–16 cycles of (98 °C for 15 s, 66 °C for 15 s, 72 °C for 10 s), and a final 72 °C for 5 min. Then, the T7-promoter-inserted library is transcripted into RNA using T7 polymerase before RNA purification using RNA clean beads (Vazyme). Then, the library RNA was then separated into two parts, with one part undergoing poly(A) tail enrichment using oligo(dT) beads (NEB, S1419S) while the other underwent rRNA depletion as previously described [12].

For C2T-seq of bulk mESC cells, about 10^6^ cells were harvested for RNA extraction using RNA purification kit (Vazyme, RC101-01). Two hundred nanograms of total RNA was used for library construction. For poly(A) selected RNA library construction, about 20 µg total RNA was used for poly(A) plus RNA enrichment by oligo(dT) coupled magnetic beads (Invitrogen, 61,005). For scm^6^A-seq of human oocytes, we performed additional poly(A) enrichment on the input, supernatant, and m^6^A-IP libraries, precisely as C2T-seq.

### RNA-seq data processing

As shown in Fig. 1b, the sequencing row data was flited according to the structure of scm^6^A-seq data using homemade script. Deduplication was performed for single-cell RNA-seq data. For cap read selection, if the libraries were constructed using smart-total kits, the adapters were trimmed, and the sequencing result was separated into 3 parts: the 4G reads (those starting with [N, G]{4,} in R2 record), 3G (those starting with [N, G]{3,3}[A, T, C]{1,} in R2 record), and internal reads (all other reads). Then, the reads were mapped to the genome using hisat2 with the parameter “-FR −5 3 −1 r2.fq.gz −2 f1.fq.gz.” Else, for scm^6^A-seq data, the adaptors were trimmed, and the sequencing result was separated into 2 parts (The 3G reads and 4G reads. Other reads were discarded), then the reads were mapped to the genome using hisat2 [57] with parameter “-FR −5 7 −1 R1.fq.gz −2 R2.fq.gz.”

For tail read selection, the reads were first separated into two parts: the read-through reads and non-read-through reads. Then, the tail reads were extracted by the R2 sequence. Those R2 reads starting with “[ATCG] {7,12} TTTTT” were identified as tail reads. For tail read mapping, the sequencing adaptors were trimmed using “cutadapt” software, and the poly(A) tail was also trimmed using the parameter “-a AAAAAAAAAA -G TTTTTTTTTT -O9 -n 10.”

### *m*^*7*^*G and m*^*6*^*A*_*m*_* identification*

The m^7^G cap reads were separated according to the non-template G. Briefly, the pre-processed reads were mapped to the genome before the m^7^G reads were extracted by the Concise Idiosyncratic Gapped Alignment Report (CIGAR) string. Those CIGAR string matched to “^[0–9] + S[1–9][0–9]{1,}M” were further filed by awk with parameter “match($6,/[0–9] + S$/){num = substr($6,RSTART,RLENGTH-1);if(substr($10,length($10)-num + 1,num) ~/^[CN] + $/){print $0}}.” For m^7^G calling, we filtered out the m^7^G sites only detected in the 4G data set. Then, the m^7^G peaks were called using PARACLU [58] according to previously reported instructions. For scm^6^A-seq data, the input, m^6^A, and supernatant data sets were merged for m^7^G peak identification. For m^6^A_m_, we identified the m^6^A_m_ peaks from the identified m^7^G cap peaks with the cutoff “A_m_ (IP) − A_m_ (input) > 0.5.”

### APA and poly(A) length analysis

For novel APA sites analysis, we extracted the mapping results from the BAM file for R1 reads, converted them into a BED file, and subsequently performed symmetric extension of the mapping records before conducting APA (alternative polyadenylation) peak analysis using MACS2 using parameter “–nomodel -p 0.05.” Then, the peaks less than 500 bp were ignored before being further filtered by the reads count in the peak regions (RPKM > 2).

For poly(A) length analysis, the pre-processed reads were mapped to the homemade reference using the 200 bp sequences around the APA site records downloaded from Genecode. Then, the 200 bp sequence record and reads were realigned using MAFFT [59] using the parameter “–reorder –quiet –auto –clustalout –localpair.” For poly(A) length analysis, the number of T in R2 reads was used for poly(A) length calculation, both the media and average number of a specific APA site were calculated, and then the APAs in the same gene region were further calculated as the length of a specific gene.

For coverage analysis of the poly(A) data around the APA sites, only the peaks in known APA sites in the reference were used for further analysis. We used deeptools computeMatrix using parameter “-b 1000 -a 1000 –binSize 1 –skipZeros” to calculate the reads count of each bin. The same number of randomly selected positions in the genome was used for background calculation.

### *m*^*6*^*A and m*^*6*^*A*_*m*_* motif enrichment analysis*

The motifs were analyzed using the online software MEME [60]. For m^6^A Motif discovery, the input sequences were extracted from the transcriptome reference. And the 200 bp sequences around the m^6^A center were used for motif enrichment. For m^6^A_m_, the 20 bp sequence after m^7^G was extracted for motif analysis.

### *CAGE-seq and Smart-seq* + *5′ data analysis*

For CAGE-seq data, reads were aligned to the genome (mm10) using HISAT2 in strand-specific mode. The first nucleotide at the 5′ end of each aligned read was identified as a potential m^7^G modification site. For Smart-seq + 5′ data, analyses were conducted following established Smart-seq + 5′ protocols. Reads were initially classified into three categories: 5′ reads, 3′ reads, and other reads. Each category was mapped independently to the reference genome. Specifically, poly(A) tails were trimmed from 3′ reads prior to genome alignment.

## Supplementary Information

Additional file 1: Supplementary Figs. 1–7. Fig. S1 A overview of coverage bias among different RNA-seq data. Fig. S2 Cap and tail analysis of C2T-seq data using C2T-APP. Fig. S3 m^6^A_m_ and m^7^G cap improves maternal RNA translation in the oocytes. Fig. S4 Maternal RNA poly-tail structure regulates translation efficiency reshaping during oocyte maturation. Fig. S5 Maternal RNA poly-tail structure regulates translation efficiency reshaping during oocyte maturation.

Additional file 2: Source data related to Figs. 1–2. Sheet “C2 T-CAGE-log2 TPM” site information and TPM values of the m^7^G sites identified using C2T-APP and CAGE-seq. Sheet “Reads-distribution” the reads coverage distribution of different methods. Sheet “mESC-multi-omics” gene expression level of mESC calculated using cap reads, tail reads, or total reads. Sheet “mESC-Tail-length-dis” tail length distribution of U-tail transcripts or total transcripts.

Additional file 3: Source data related to Fig. 3. Sheet “M3 WT-KO-GV-noGap-matrix” single cell gene reads count matrix of GV oocytes calculated using non-cap reads. Sheet “M3 WT-KO-GV-Gap-matrix” single cell reads count matrix of GV oocytes calculated using cap reads. Sheet “M3 WT-KO-GV-m6 m-matrix” single cell reads count matrix of GV oocytes calculated using m^6^A_m_ reads. Sheet “M3 WT-KO-metaData” the sample information of scm^6^A-seq data.

Additional file 4: Source data related to Fig. 4. Sheet “GV-MII-CHX-cap” gene expression level of GV, MII, and CHX treated MII calculated using cap reads. Sheet “GV-MII-CHX-genebody” gene expression level of GV, MII, and CHX treated MII calculated using non-cap reads. Sheet “GV-MII-CHX-tail” gene expression level of GV, MII, and CHX treated MII calculated using tail reads.

Additional file 5: Source data related to Fig. 5. Sheet “cap” gene reads count of individual human oocytes calculated using cap reads. Sheet “GeneBody” gene reads count of individual human oocytes calculated using non-cap reads. Sheet “polyA” gene reads count of individual human oocytes calculated using polyreads. Sheet “m6 Am” gene reads count of individual human oocytes calculated using m^6^A_m_ reads. Sheet “m6 A” gene reads count of individual human oocytes calculated using m^6^A reads. Sheet “TailLength” polylength of each APA sites of individual human oocytes calculated using m^6^A reads.

Additional file 6. A comprehensive result of the polystructure of mESC cell line, the result file is an original output of C2T-APP.

Additional file 7. Excel file contains raw individual data pointsfor each figure. Data are organized into separate worksheets, with each sheet corresponding to a specific figure.

## Data Availability

All data generated or analysed during this study are included in this published article, its supplementary information files and publicly available repositories. The raw sequencing data generated in this study have been deposited in the Genome Sequence Archive of National Genomics Data Center (https://ngdc.cncb.ac.cn/) under accession code CRA017522, CRA023822, HRA007855 and HRA007856. The scm6 A data was downloaded from National Genomics Data Center [61] under accession code PRJCA008779, the SMARTer Stranded Total RNA-Seq data [62] was downloaded under the accession code CRR274750. The GAGE-seq data [63, 64] were downloaded from the Sequence Read Archive database under accession code SRR7300113 and SRR7300114. For the Smart-seq + 5’ data [65, 66], the accession codes are SRR23403774 and SRR23403777. For the Ribo-seq data [67], the accession code is SRR13577576, SRR13577577, SRR13577580, SRR13577581, SRR13577582, SRR13577583, SRR18677119 and SRR18677120. The Proteome data [68] was downloaded from the published supplementary data. The matrix data generated in this study are provided in the Supplementary Information and Source Data files (Additional file 2–7). Source data has also been uploaded to Figshare and can be accessed via the following https://doi.org/10.6084/m9.figshare.26264855. The original image data are available on Mendeley Data under the https://doi.org/10.17632/g9r987v9j4.1.

## Abbreviations

ART: Assisted reproductive technology
RNA-seq: RNA sequencing
scm6 A-seq: Single-cell m6A sequencing
C2T-APP: Cap to Tail sequencing application
C2T-seq: Cap to Tail sequencing
m6 A: N6-methyladenosine
m6 Am: N6, 2′-O-dimethyladenosine
m7G: N7-methylguanosine
APA: Alternative polyadenylation
GV: Germinal vesicle
MII: Metaphase II
TSO: Template switching oligo
TSS: Transcription start site
WES: Whole exome sequencing
mESC: Mouse embryonic stem cell
IVM: In vitro maturation
CAGE-seq: Cap Analysis Gene Expression sequencing
CHX: Cycloheximide
MACS2: Model-based Analysis of ChIP-Seq
MAFFT: Multiple Alignment using Fast Fourier Transform
ATAC-seq: Assay for Transposase-Accessible Chromatin using sequencing
RNA-IP: RNA immunoprecipitation
rRNA: Ribosomal RNA
PCR: Polymerase chain reaction
RT: Reverse transcription
PE150/PE250: Paired-End 150/250 bp sequencing
