# Supplementary material for: RNA multi-omics in single cells reveal rhythmical RNA reshaping during human and mouse oocyte maturation
Source: BMC Biol. 2025 May 28;23:147. doi: 10.1186/s12915-025-02250-7 (PMC12121112; doi:10.1186/s12915-025-02250-7)
Supplement: Supplementary file 1 — Additional file 1: Supplementary Figs. 1–7. Fig. S1 A overview of coverage bias among different RNA-seq data. Fig. S2 Cap and tail analysis of C2T-seq data using C2T-APP. Fig. S3 m6Am and m7G cap improves maternal RNA translation in the oocytes. Fig. S4 Maternal RNA poly-tail structure regulates translation efficiency reshaping during oocyte maturation. Fig. S5 Maternal RNA poly-tail structure regulates translation efficiency reshaping during oocyte maturation. [file 12915_2025_2250_MOESM1_ESM.pdf]

RNA-multi-omics in single cells reveal rhythmical RNA reshaping during human and mouse oocyte maturation

Huan Yao[1,2†*], Danru Zhang[3†], Haixia Jin[1†], Yanjie Guo[1†], Yan Liu[1], Shengnan Wang[1], Tong Li[1], Shenli Yuan[2], Gang Lu[2*], Yingpu Sun[1*]

†Huan Yao, Danru Zhang, Haixia Jin, and Yanjie Guo contributed equally to this work

*Correspondence:

Huan Yao
huanyao@cuhk.edu.hk,
Gang Lu
lugang@cuhk.edu.hk
Yingpu Sun
syp2008@vip.sina.com

[1]Center for Reproductive Medicine, Henan Key Laboratory of Reproduction and Genetics, The First Affiliated Hospital of Zhengzhou University, Zhengzhou, China

[2]CUHK-SDU Reproductive Genetics Joint Laboratory, School of Biomedical Sciences, The Chinese University of Hong Kong, Hong Kong

[3]CAS Key Laboratory of Genomic and Precision Medicine, Beijing Institute of Genomics, Chinese Academy of Sciences and China National Center for Bioinformation

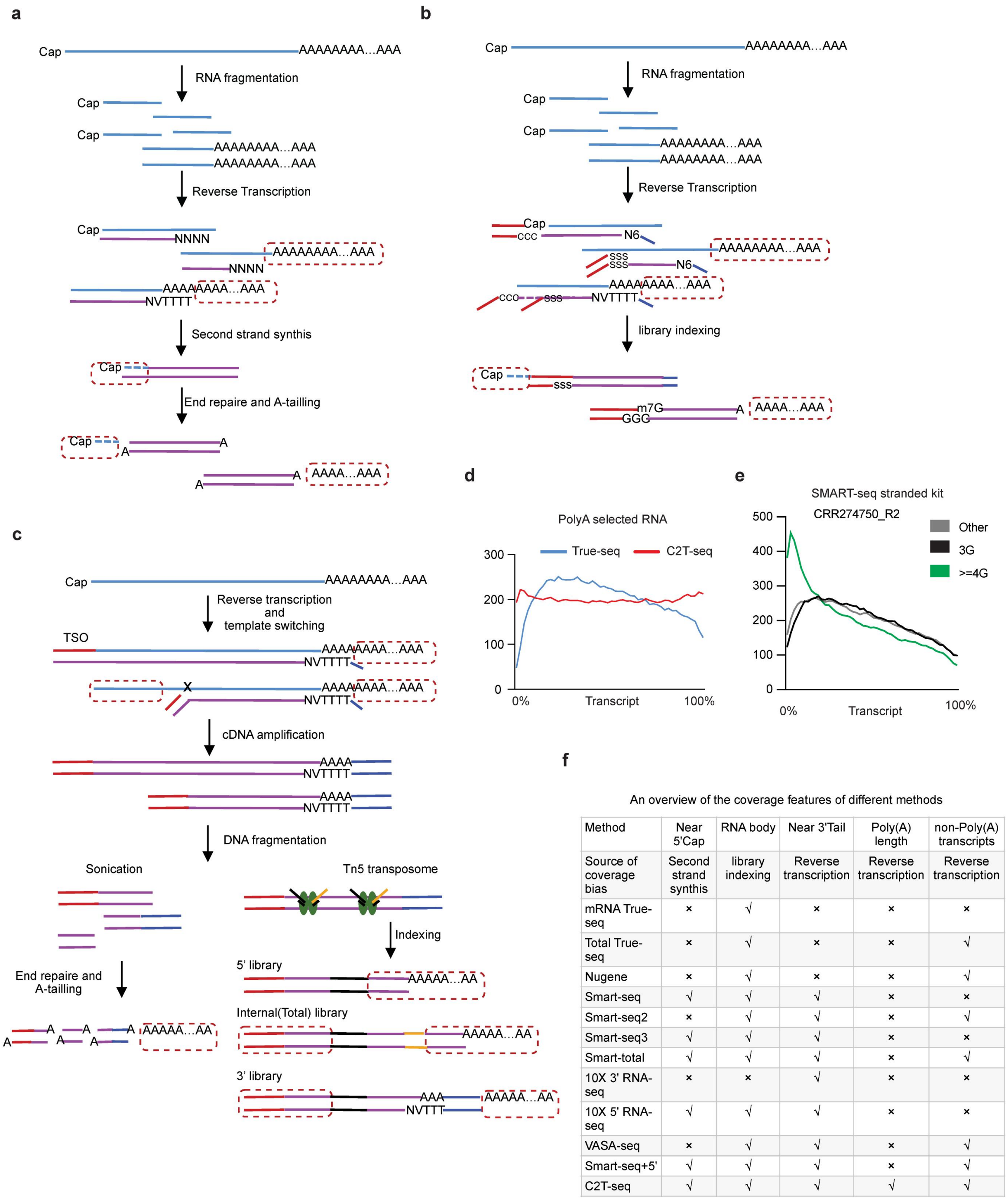

Method	Near 5'Cap	RNA body	Near 3'Tail	Poly(A) length	non-Poly(A) transcripts
Source of coverage bias	Second strand synthis	library indexing	Reverse transcription	Reverse transcription	Reverse transcription
mRNA True-seq	×	√	×	×	×
Total True-seq	×	√	×	×	√
Nugene	×	√	×	×	√
Smart-seq	√	√	√	×	×
Smart-seq2	×	√	√	×	√
Smart-seq3	√	√	√	×	×
Smart-total	√	√	√	×	√
10X 3' RNA-seq	×	×	√	×	×
10X 5' RNA-seq	√	√	√	×	×
VASA-seq	×	√	√	×	√
Smart-seq+5'	√	√	√	×	√
C2T-seq	√	√	√	√	√

Figure S1| A overview of coverage bias among different RNA-seq data. a-c Schematic diagram showing the the missing RNA elements during standard RNA-seq protocol (**a**), SMART-total RNA-seq protocol (**b**), and SMART-seq protocol (**c**), the missing RNA elements are marked with a red dashed box. **d** A summary of the coverage bias of some popular existing RNA-seq methods and C2T-seq. **d** Meta-gene plot showing the coverage distribution of libraries

constructed using True-seq kit (TAKARA) and C2T-seq using poly(A) selected mRNA. **e** Meta-gene plot showing the coverage of different classes of reads, the reads are classified using the R1 reads of SMART-total libraries. **f** A summary of the coverage bias of some popular existing RNA-seq methods and C2T-seq.

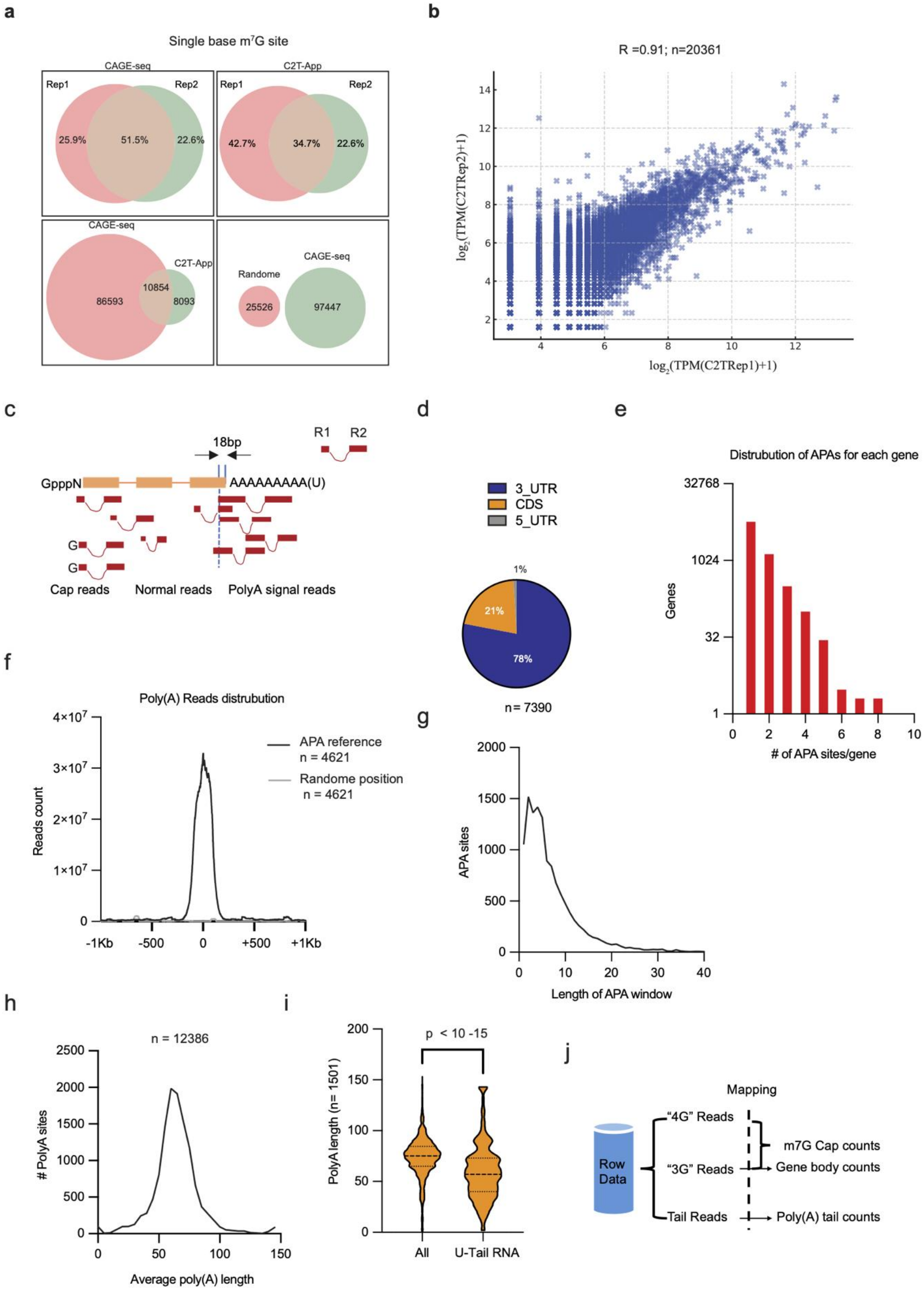
a
Single base m7G site
CAGE-seq
Rep1
Rep2
25.9%
51.5%
22.6%
C2T-App
Rep1
Rep2
42.7%
34.7%
22.6%
CAGE-seq
C2T-App
86593
10854
8093
Randome
CAGE-seq
25526
97447
b
R =0.91; n=20361
log2(TPM(C2TRep2)+1)
log2(TPM(C2TRep1)+1)
c
18bp
R1
R2
GpppN
AAAAAAAAA(U)
G
G
Cap reads
Normal reads
PolyA signal reads
d
3_UTR
CDS
5_UTR
1%
21%
78%
n= 7390
e
Distrubution of APAs for each gene
Genes
# of APA sites/gene
f
Poly(A) Reads distrubution
Reads count
APA reference
n = 4621
Randome position
n = 4621
-1Kb
-500
0
+500
+1Kb
g
APA sites
Length of APA window
h
n = 12386
# PolyA sites
Average poly(A) length
i
p < 10 -15
PolyA length (n= 1501)
All
U-Tail RNA
j
Mapping
Row Data
"4G" Reads
"3G" Reads
Tail Reads
m7G Cap counts
Gene body counts
Poly(A) tail counts

Figure S2| Cap and Tail analysis of C2T-seq data using C2T-App. a Venn plots illustrating the overlap of single-base m^7G sites identified by two replicate experiments using CAGE-seq (upper left) and C2T-App (upper right). Lower panels show overlaps between m^7G sites identified by CAGE-seq and C2T-App (bottom left), and between sites identified by CAGE-seq and randomly selected sites (bottom right). **b** Scatter plot showing the correlation of m^7G TPM values between two replicates of C2T-App (R = 0.91, n = 20361). **c** Schematic diagram showing the genome mapping result of cap reads, Normal reads (Gene body reads), and tail reads. **d** Pie Chat showing the distribution of APA sites identified using poly(A) reads. **e** Bar chat showing the distribution of the number of detected APA sites for each gene. **f** Density plot displaying the distance between identified APA peaks using poly(A) reads and annotated APA sites (red) or randomly selected sites (grey). **g** Density plot of the length of APA windows. **h** Density plot of the poly(A) length of each APA sites (n = 12386). **i** Violin plot showing the poly(A) tail length of all Poly(A) reads, or U-tail reads of uridylation medicated genes (n= 1501). **j** A simplified illustration of C2T-APP workflow.

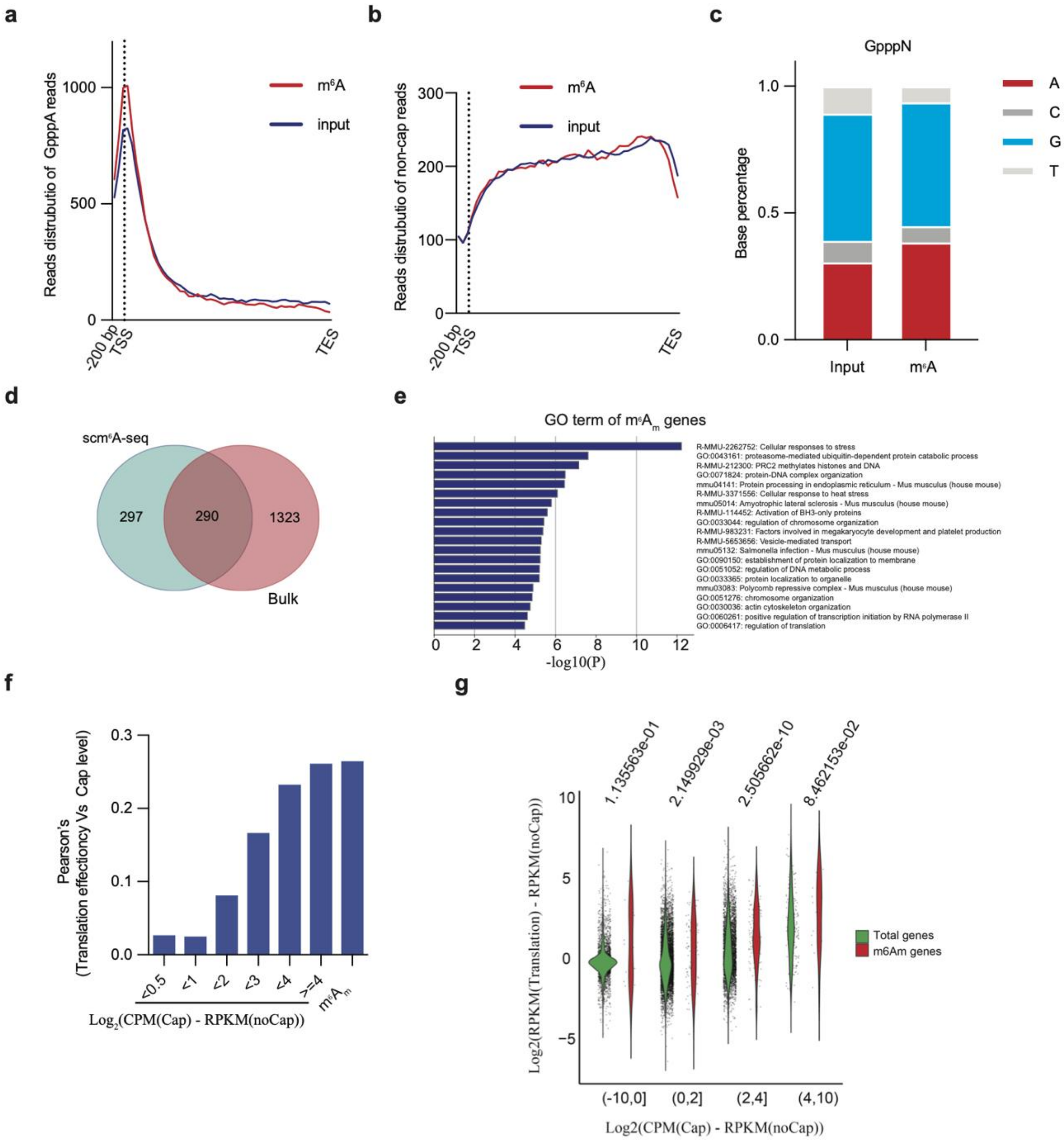

Figure S3| m^6A_m and m^7G cap improves maternal RNA translation in the oocytes. a-b Meta-gene plot showing the coverage distribution of m^7G cap reads (**a**) and non-cap reads (**b**) in input and m^6A-IP data sets. **c** The first base neat the m^7G cap in input and m^6A-IP data sets. **d** Venn plot showing the detected m^6A_m genes in scm^{6}A-seq data and bulk m^6A-seq data of mouse oocytes. **e** Go analysis result of the common m^6A_m genes of mouse oocytes. The analysis was performed using Metascape using default parameters. **f** Bar plot showing the correlation between translation and m^7G cap level. The gene sets were separated into different classes according to the m^7G cap level. **g** Violin plot showing the different translation efficiency between m6Am genes and other genes. The gene sets were separated into different classes according to the m^7G cap level. The two-sided P value was calculated by unpaired student t-test.

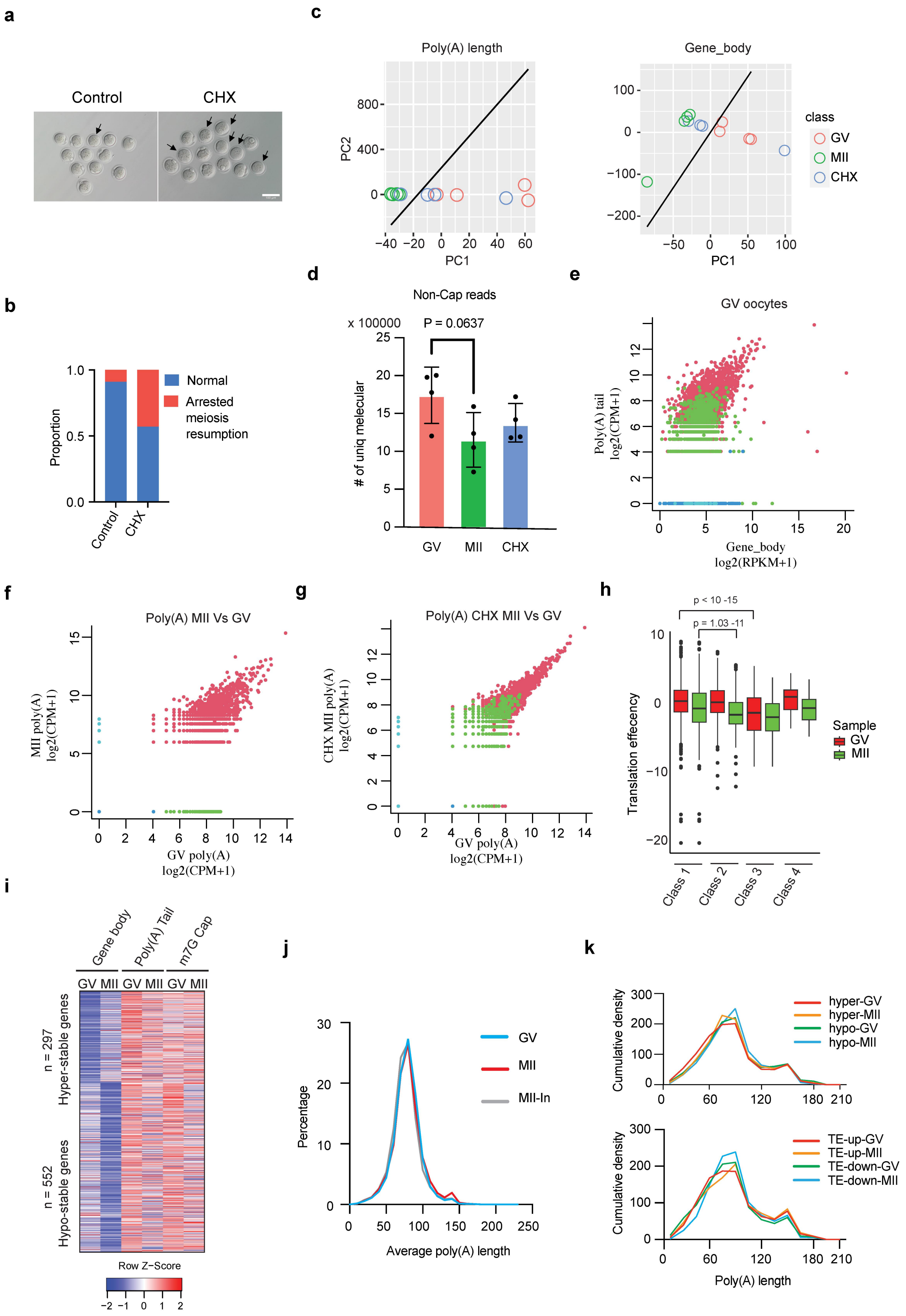
a
Control
CHX
b
Proportion
Normal
Arrested meiosis resumption
Control
CHX
c
Poly(A) length
Gene_body
PC1
PC2
class
GV
MII
CHX
d
Non-Cap reads
x 100000
P = 0.0637
# of uniq melecular
GV
MII
CHX
e
GV oocytes
Poly(A) tail log2(CPM+1)
Gene_body log2(RPKM+1)
f
Poly(A) MII Vs GV
MII poly(A) log2(CPM+1)
GV poly(A) log2(CPM+1)
g
Poly(A) CHX MII Vs GV
CHX MII poly(A) log2(CPM+1)
GV poly(A) log2(CPM+1)
h
p < 10 -15
p = 1.03 -11
Translation effecency
Sample
GV
MII
Class 1
Class 2
Class 3
Class 4
i
Gene body
Poly(A) Tail
m7G Cap
GV MII GV MII GV MII
n = 297 Hyper-stable genes
n = 552 Hypo-stable genes
Row Z-Score
j
Percentage
GV
MII
MII-In
Average poly(A) length
k
Cumulative density
hyper-GV
hyper-MII
hypo-GV
hypo-MII
TE-up-GV
TE-up-MII
TE-down-GV
TE-down-MII
Poly(A) length

Figure S4| Maternal RNA Poly(A)-tail structure regulates translation efficiency reshaping during oocyte maturation. **a** In vitro maturation (IVM) result of mouse oocytes in both the control (n=12) and CHX treated group (n=14), the arrested oocytes were labeled using black arrows. **b** Bar plot showing the proportion of cell types of IVM result. **c** Dot plot showing the PCA matrix of poly(A) length data (left) and gene body data (right) of GV oocytes, MII oocytes, and CHX-treated MII oocytes. The line shows the SVM decision function calculated using normal GV oocytes and MII oocytes. **d** Bar plot showing the non-cap (gene body) reads detected in the GV oocytes (n=4), MII oocytes (n=4), and CHX-treated MII oocytes (n=4). The two-sided P value was calculated by unpaired student t-test. **e-g** Dot plot showing the gene expression level of poly(A)ome expression level and gene body expression level in GV oocytes, the gene expression level of poly(A)ome in GV oocytes and MII oocytes (**f**) and the gene expression level of poly(A)ome in normal MII oocytes and MII oocytes treated with CHX (**g**). The different gene classes are colored in different colors. **h** Bar plot showing the translation efficiency of different gene sets in GV oocytes and MII oocytes. The two-sided P value was calculated by unpaired student t-test. **i** Heatmap showing the multi-omics expression level (gene-body, poly(A), m^7G) of hyper-stable genes and hypo-stable genes in GV oocytes and MII oocytes. **j** Density plot showing the distribution of poly(A) length in GV oocytes, MII oocytes, and CHX-treated MII oocytes. **k** Density plot showing the distribution of poly(A) length in different gene sets, the genes are classified according to RNA stability (upper) and translation efficiency (lower).

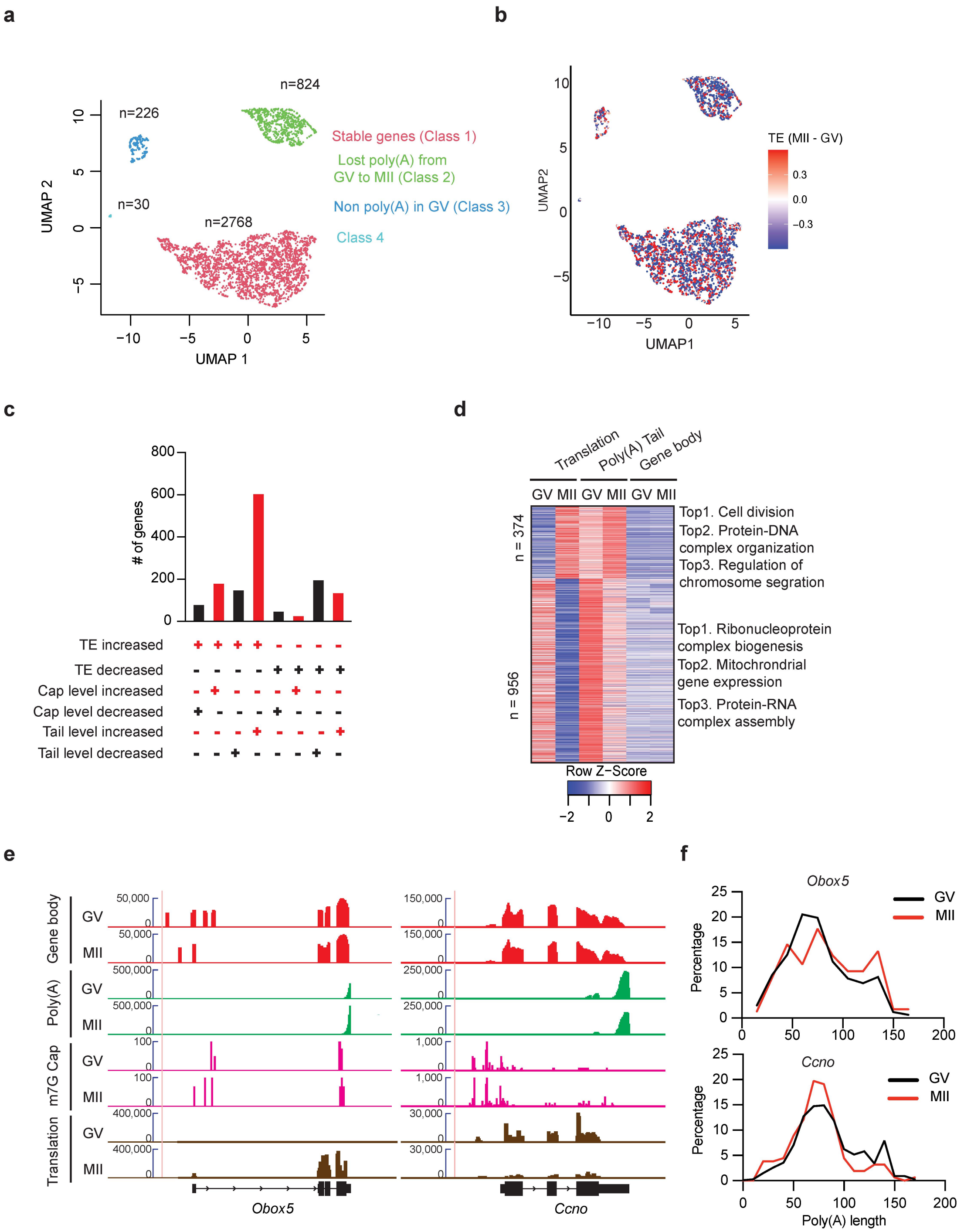

Figure S5| Maternal RNA Poly(A)-tail structure regulates translation efficiency reshaping during oocyte maturation. a-b UMAP showing the different gene sets metabolized differently during oocyte maturation and the genes were classified into four classes (**a**). The translation efficiency changes of each gene were plotted in different colors (**b**). **c** Upset plot showing the

relationship among translation efficiency, cap level, and tail level changes during oocyte maturation. **d** Heat map showing the presentative translation efficiency increased genes and translation efficiency decreased genes. The poly(A) gene expression level and gene body expression level of these presentative genes are shown. The top there results of the GO analysis are listed beside. **e** Integrated Genomics Viewer (IGV) diagram showing the translation level, m^7G cap, poly(A), and gene body expression level of *Obox5* and *Ccno* genes expression in GV and MII oocytes. **f** Density plot showing the distribution of the poly(A) length of *Obox5* and *Ccno*.

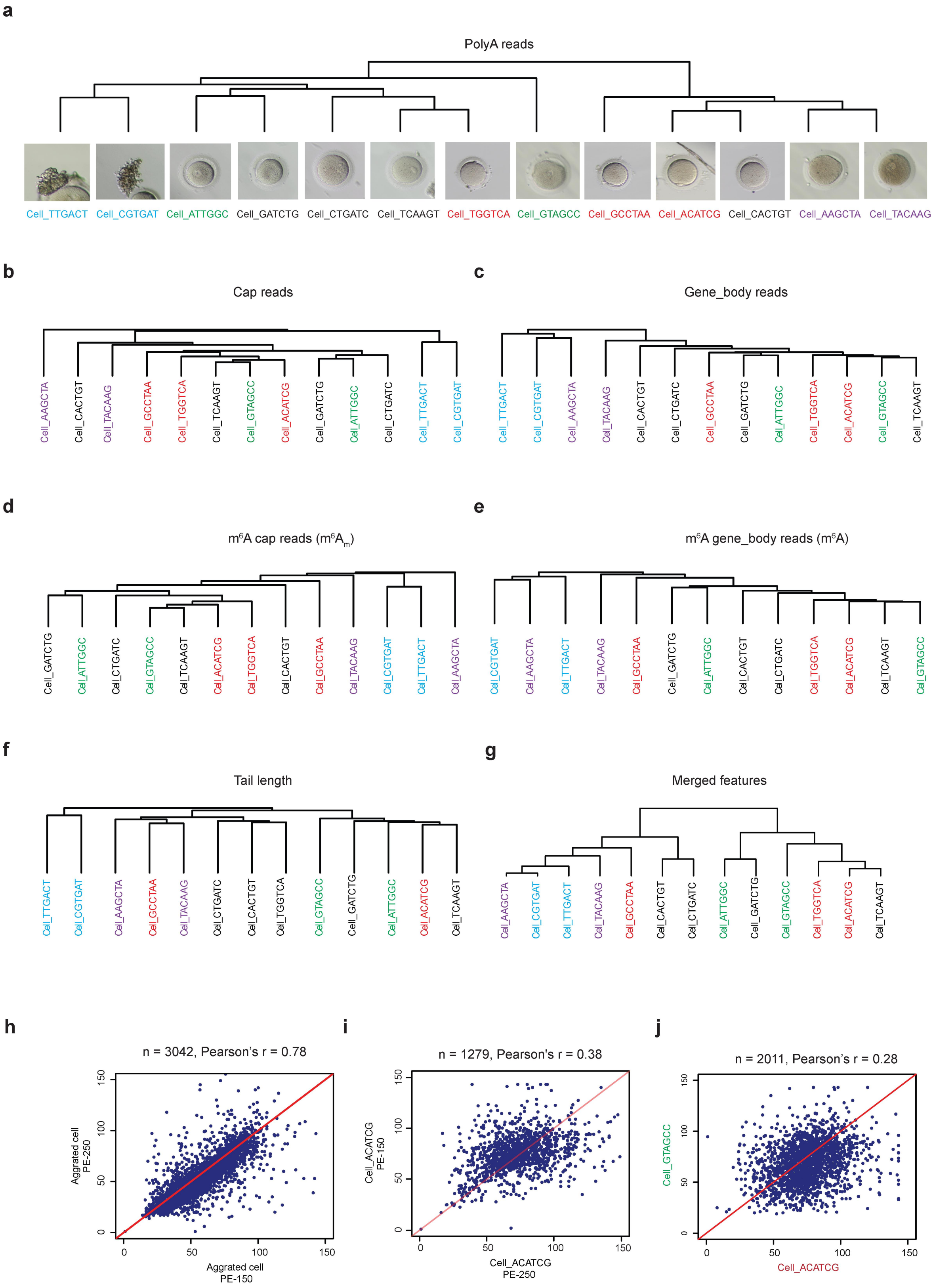
a
PolyA reads
Cell_TTGACT Cell_CGTGAT Cell_ATTGGC Cell_GATCTG Cell_CTGATC Cell_TCAAGT Cell_TGGTCA Cell_GTAGCC Cell_GCCTAA Cell_ACATCG Cell_CACTGT Cell_AAGCTA Cell_TACAAG
b
Cap reads
c
Gene_body reads
d
m6A cap reads (m6Am)
e
m6A gene_body reads (m6A)
f
Tail length
g
Merged features
h
n = 3042, Pearson's r = 0.78
Aggrated cell PE-250
Aggrated cell PE-150
i
n = 1279, Pearson's r = 0.38
Cell_ACATCG PE-150
Cell_ACATCG PE-250
j
n = 2011, Pearson's r = 0.28
Cell_GTAGCC
Cell_ACATCG

Figure 6| Maternal RNA Poly(A)-tail structure regulates translation efficiency reshaping during oocyte maturation. **a-g** Hierarchical clustering of single oocytes using the feature of poly(A) structure level (**a**), m^7G cap level (**b**), gene expression level (**c**), m^6A_m level (**d**), m^6A level (**e**), tail length (**f**) and merged PCA matrix data (**g**). **h-i** Dot plot showing the correlation between poly(A) tail length result of each gene calculated using the PE150 or PE250 data. Both aggregated data (**h**) and single-cell data (**i**) are shown. **j** Dot plot showing the correlation of poly(A) tail length result of each gene between different cells
